# Maximal oxygen consumption increases with temperature in the European eel (*Anguilla anguilla*) through increased heart rate and arteriovenous extraction

**DOI:** 10.1093/conphys/cow027

**Published:** 2016-08-26

**Authors:** Débora Claësson, Tobias Wang, Hans Malte

**Affiliations:** Department of Bioscience, Zoophysiology, Aarhus University, C. F. Møllers Allé 3, DK08000 Aarhus C, Denmark

**Keywords:** Aerobic scope, blood flow, heart rate, oxygen- and capacity-limited thermal tolerance, oxygen consumption, temperature

## Abstract

Global warming results in increasing water temperature, which may represent a threat to aquatic ectotherms. The rising temperature affects ecology through physiology, by exerting a direct limiting effect on the individual. The mechanism controlling individual thermal tolerance is still elusive, but some evidence shows that the heart plays a central role, and that insufficient transport of oxygen to the respiring tissues may determine the thermal tolerance of animals. In this study, the influence of the heart in thermal limitation was investigated by measurements of aerobic scope in the European eel (*Anguilla anguilla*) together with measurements of cardiac output during rest and activity. Aerobic capacity was not limited by an acutely increased temperature in the European eel. Oxygen demand was met by an increase in heart rate and arteriovenous extraction. These findings suggest that thermal tolerance during exposure to acute temperature changes is not defined by oxygen transport capacity in the eel, and other mechanisms may play a central role in limiting thermal tolerance in these fish.

## Abstract (precis)

We measured oxygen consumption, blood flow and heart rate at rest and at maximum activity in eels exposed to different temperatures. We found that, although the ability to increase heart rate in response to heavy exercise was reduced at temperatures close to the upper critical temperature, this did not limit the oxygen consumption.

## Introduction

The current shifts in phenology, distribution and abundance of aquatic ectotherms have been correlated with direct effects of rising temperatures on bodily functions, and future conservation strategies, therefore, depend on an ability to understand how temperature affects physiological processes at the organism level (Pörtner, 2012; Schulte, 2015). In fishes and other ectotherms, elevated temperature increases the standard metabolic rate (SMR), measured as the minimal oxygen consumption (${\dot{M}}_{O_{2}}$) of inactive and post-absorptive animals that are not recovering from anaerobic exercise (Fry and Hart, 1948). The difference between the maximal oxygen consumption (${\dot{M}}_{O_{2}\max}$) and SMR is defined as the aerobic scope (AS; Fry and Hart, 1948), and this capacity is used extensively to assess potential impacts of climate change on fishes (Pörtner and Knust, 2007; Wang and Overgaard, 2007; Pörtner and Farrell, 2008).

The so-called ‘oxygen- and capacity-limited thermal tolerance (OCLTT) model’ states that the failure of oxygen transport systems to match bodily oxygen demand dictates thermal tolerance (Pörtner and Knust, 2007; Pörtner and Farrell, 2008). This model predicts that performance quickly deteriorates above the optimal temperature for AS (*T*_optAS_) as a result of the inability of the oxygen transport systems to cope with the higher oxygen demand (Frederich and Pörtner, 2000; Pörtner, 2010). As a non-exclusive alternative, the temperature tolerances of physiological and biochemical capacities have co-evolved, so that the *T*_optAS_ coincides with the temperature at which performance (such as locomotion and growth) and fitness (i.e. survival and reproductive success) are optimal (e.g. Clark *et al*., 2013). In this case, limited oxygen delivery is not the mechanistic cause for the critical thermal maximum (CT_max_).

Any component of the oxygen transport cascade responsible for bringing oxygen from the water to the mitochondria may limit ${\dot{M}}_{O_{2}\max}$, but given that arterial oxygen levels normally remain high, most studies emphasize the convective transport of oxygen in the blood as a limitation (Kiceniuk and Jones, 1977; Wang and Malte, 2011). Cardiac output ($\dot{Q}$) can be increased through elevations of stroke volume (*V*_s_) and/or heart rate (*f*_H_), both of which depend on adequate oxygen supply to the cardiac muscle. In fishes, the myocardial oxygen delivery may be limited at high temperatures, because the spongy myocardium is devoid of coronary perfusion; hence, it depends entirely on oxygen availability in the oxygen-poor, venous blood. We hypothesize that AS decreases above *T*_optAS_ in concert with a gradual collapse in $\dot{Q}$. To investigate this, ${\dot{M}}_{O_{2}}$ and ${\dot{M}}_{O_{2}\max}$ were measured over a broad temperature range in European eels (*Anguilla anguilla*). The eel provides a good model for investigating cardiac variables, owing to their easily exposable ventral aorta. With a flow probe placed around the ventral aorta, $\dot{Q}$ and *f*_H_ were quantified at increasing temperatures. Thus, the question of whether there was a collapse in the cardiac function in European eels with the increasing temperatures could be addressed.

## Materials and methods

### Experimental animals

European eels (*A. anguilla*) of undetermined sex (299 ± 84 g) were purchased from Lyksvad fish farm (Vamdrup, Denmark) and kept at Aarhus University for no less than 3 weeks in normoxic [partial pressure of oxygen ($P_{O_{2}}$) >140 mmHg] and non-chlorinated tap water at 18°C, with a photoperiod of 12 h light–12 h dark. The water was recirculated and biologically filtered (Akva Group, Vejle, Denmark) at a flow of 1000 l/h, and the temperature and oxygen were monitored continuously. The eels were fed ~0.7% body mass/day with Dan-Ex eel pellets (Biomar A/S, Brande, Denmark), but fasted for at least 48 h before experiments. All eels were tagged with FDX-B Passive Integrated Transponder (PIT tag from Loligo Systems, Tjele, Denmark) inserted through a 5 mm incision in the ventral body wall under immersion anaesthesia (0.5 g/l benzocaine). All experiments were approved by the Danish Animal Experiments Inspectorate (permit no. 2012-15-2934-00246).

### Determination of the critical thermal maximum

The CT_max_ was estimated as the temperature at which the eels began to lose equilibrium. Eight eels (311 ± 19 g) were placed in a 135 litre container filled with aerated freshwater at 18°C. After 1 h, the temperature was increased at 1.8°C h^−1^ using a Julabo FP51 cooler. When the eel was unable to maintain equilibrium, temperature was registered, and it was immediately transferred to fully aerated water for recovery at 18°C.

### Measurements of standard metabolic rate and maximal oxygen uptake

The ${\dot{M}}_{O_{2}}$ was measured in 41 eels (324 ± 10 g) immediately after enforced activity and subsequent rest using intermittent closed respirometry (Steffensen, 1989). This method and protocol provides robust measures of maximal metabolic (aerobic) rate (MMR) and SMR in inactive and resting fish species (Clark *et al*., 2013). A 135 litre tank was filled with aerated freshwater and connected to a Heto HMT 200 thermostat to maintain temperatures within ±0.5°C. The fish were enclosed in submerged respirometers (2.5 litres) where a galvanic oxygen electrode (Oxyguard mini connected to a Loligo Systems Loli-DAQ data acquisition box), calibrated daily in anoxic and fully aerated water, measured the decline in water $P_{O_{2}}$ at 1 Hz. Water constantly circulated past the electrode at a steady flow, so ${\dot{M}}_{O_{2}}$ could be calculated from the slope of linear regression of $P_{O_{2}}$ vs. time (in kilopascals per minute) using the following equation:

$${\dot{M}}_{O_{2}}=\frac{\beta_{O_{2}}V}{M_{b}}\times \frac{\Delta P_{O_{2}}}{\Delta t},$$

where $\Delta P_{O_{2}}/\Delta t$ is the change in water oxygen pressure per unit time, β is the oxygen solubility in water, *V* is the volume of the respirometer, and *M*_b_ is the body mass of the fish.

At each test temperature, the duration of the closed periods was adjusted differently to ensure that $P_{O_{2}}$ never fell below 18 kPa. The tank was connected to an ultraviolet filter to reduce bacterial growth. Throughout the subsequent 48 h, the tank was shielded to minimize visual disturbance during measurements of SMR. The system was automated, and after each measurement the respirometers were flushed for 200 s to replenish O_2_ and get rid of CO_2_ and other excretion products. The order of test temperatures and the eel used were randomized to minimize time bias. At the end of each experiment, bacterial ${\dot{M}}_{O_{2}}$ was measured in the empty respirometers for 1 h, and the value, which never exceeded 10% of the fish, was subtracted. The respirometer and all tubing were carefully cleaned before the next experiment. Data were analysed using a Mathematica script (version 5.2; Wolfram Research, Champaign, IL, USA).

Eels were quickly transported from the holding tank to the laboratory in water from the holding tank. Before introducing the eels to the respirometers, they were transferred to a container with water at the experimental temperature and exercised by chasing. The container was ellipsoidal and allowed for burst-and-glide swimming. One eel was transferred to the container at a time and left for 10 min before the chasing was commenced. Chasing was continued until the eels no longer responded to tactile stimuli and appeared exhausted. This procedure is suitable for an ambush predator, such as the eel, that does not undertake long periods of swimming in their freshwater cycle (Schultz *et al*., 2009). As a consequence, the critical swimming speed (*U*_crit_) protocol (Brett, 1964) would be unlikely to elicit MMR in eels (Peake and Farrell, 2006). After exhaustion, the eels were quickly returned to the respirometers, and the measurement was started immediately.

The ${\dot{M}}_{O_{2}\max}$ was considered to be the highest ${\dot{M}}_{O_{2}}$ measurement, which normally occurred during the first measurement of O_2_ uptake after chasing. The SMR was estimated as the mean of the 10% lowest ${\dot{M}}_{O_{2}}$ values excluding outliers (>2 SD from the mean).

### Measurements of the cardiovascular responses to exercise at various temperatures

Eels were anaesthetized by immersion in freshwater containing benzocaine (0.5 g/l) until ventilation ceased. Next, the eels were placed on an operating table, where their gills could be irrigated with aerated freshwater containing benzocaine (0.1 g/l). Xylocaine (0.3 ml, 20 mg/ml) was injected subcutaneously before a 1 cm ventral mid-line incision allowed a Transonic flowprobe to be placed around the ventral aorta. The incision was closed with three sutures, and the probe lead was fixed ventrally on the skin with stitches. For recovery, the eels were placed in individual 10 litre restrainers contained in a 120 litre aquarium with aerated freshwater at 18°C. The procedure took <30 min, and the first measurements were taken 24 h after surgery. After recovery, the water temperature was acutely changed to 10, 20 or 28°C by resetting the thermostat. To assist cooling, ice was added in the thermostat until the aquarium temperature was close to the target.

Heart rate was derived from the pulsatile flow measurements. Data were recorded at 200 Hz using an MP100 data acquisition system (Biopac Systems Inc., Goleta, CA, USA), connected to a computer running AcqKnowledge 3.9.1 (Biopac Systems Inc.). To avoid the influence of disturbance on the resting measurements, data sampling started no earlier than 15 min after the probe was connected to the system. Then, the probe was disconnected from the flowmeter, and the eels were activated by chasing to exhaustion for activity measurements. At exhaustion, eels were no longer able to maintain equilibrium and were placed back in the resting chamber, and the probe was reconnected to the flowmeter. After completion of each measurement, one-third of the water in the tank was replaced with freshwater to minimize waste products. Immediately after the experiment, the eels were killed by an overdose of benzocaine followed by decapitation.

Heart rate and $\dot{Q}$ were assessed over 200 consecutive beats, and *V*_s_ was derived from $\dot{Q}$ and *f*_H_. The Fick equation was applied for calculations of arteriovenous difference, as follows:

$${\dot{M}}_{O_{2}}=\dot{Q}(C_{{aO}_{2}}-C_{{\bar{v}O}_{2}}),$$

where $\left(C_{{aO}_{2}}-C_{{\bar{v}O}_{2}}\right)$ is the arteriovenous oxygen concentration difference.

### Statistics

Statistical analyses were performed with the software SigmaPlot^®^ 11.0 (Systat Software Inc., San Jose, CA, USA). One-way analysis of variance (ANOVA) was performed for ${\dot{M}}_{O_{2}}$, $\dot{Q}$, *f*_H_ and *V*_s_, with temperature as an independent factor. In *post hoc* multiple comparisons, Holm–Sidak correction was used. Data that did not pass the normality and equal variance tests were log transformed. Dunn's test was applied for non-parametric analysis. Student's paired *t*-test was applied when testing for significant differences between rest and activity at the same temperature. All data were tested for homogeneity of variance and normality before parametric tests. Differences were considered significant when *P* < 0.05. Data are presented as means ± SEM.

## Results

### Acute thermal tolerance

The highest water temperature tolerated by the eels was 31.5 ± 0.2°C, at which they attempted to escape, followed by clear signs of equilibrium loss. It was, however, also clear that the fish did not tolerate prolonged exposure to 30°C, indicating that the ecologically relevant CT_max_ is lower than that revealed by acute exposure to elevated temperatures. All eels quickly recovered upon return to cooler water.

### Oxygen consumption at rest and during activity

**Figure 1:**
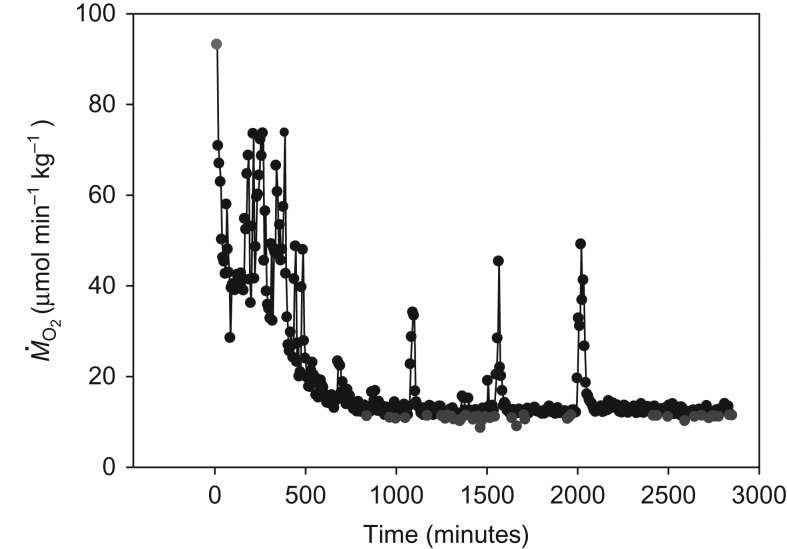
Representative oxygen consumption (${\dot{M}}_{O_{2}}$) measurement at 20°C. The red dot represents maximal metabolic rate, measured immediately after chasing, and blue dots indicate 10% of the lowest ${\dot{M}}_{O_{2}}$ data points.

The recovery of ${\dot{M}}_{O_{2}}$ upon chasing typically resembled the example shown in Fig. 1, where ${\dot{M}}_{O_{2}}$ was high immediately after exercise and then returned to a basal level interrupted by short bouts of spontaneous activity that caused brief elevations of ${\dot{M}}_{O_{2}}$. In all individuals, it was straightforward to identify SMR, as shown by the blue symbols in Fig. 1.

**Figure 2:**
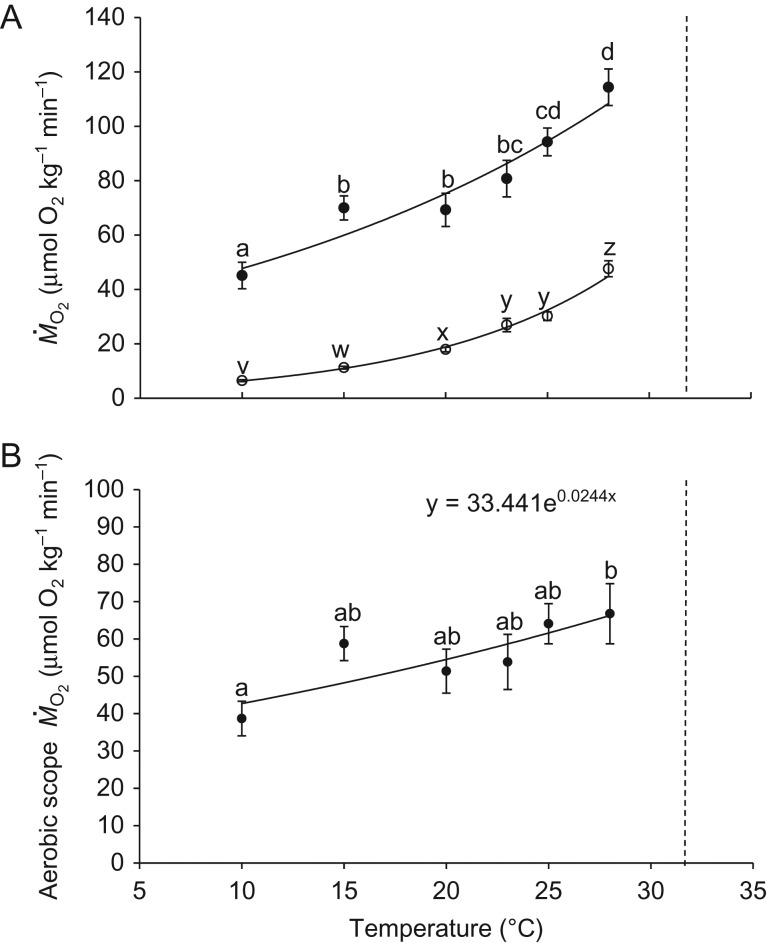
(**A**) Standard metabolic rate (SMR; open circles) and maximal metabolic rate (MMR; filled circles) at different temperatures for eels acclimated to 18°C. The SMR and MMR increased with temperature over the temperature range tested here. The increases in SMR and MMR fitted exponential lines reasonably well (*R*^2^ = 0.99 and *R*^2^ = 0.92, respectively). Different letters indicate significant differences between temperatures. Data are means ± SEM; *n* = 6–9. (**B**) Aerobic scope with increasing temperatures. The dashed line represents the critical thermal maximum (CT_max_). Different letters indicate significant differences between temperatures. Data are means ± SEM; *n* = 6–9.

Both ${\dot{M}}_{O_{2}}$ and ${\dot{M}}_{O_{2}\max}$ increased with temperature (*P* < 0.005 for all significant groups; one-way ANOVA; Fig 2A). The SMR increased almost exponentially, with a *Q*_10_ of 2.95 (*y* = 2.1618e^0.1083*x*^; *R*^2^ = 0.99). It was not possible to measure ${\dot{M}}_{O_{2}}$ at or above 30°C because prolonged exposure to this temperature resulted in equilibrium loss. Aerobic scope tended to increase with elevated temperature (*P* < 0.003; one-way ANOVA; Fig. 2B), and there were no sign of a peak in AS at an intermediate temperature.

### Cardiovascular responses to exercise and temperature

**Figure 3:**
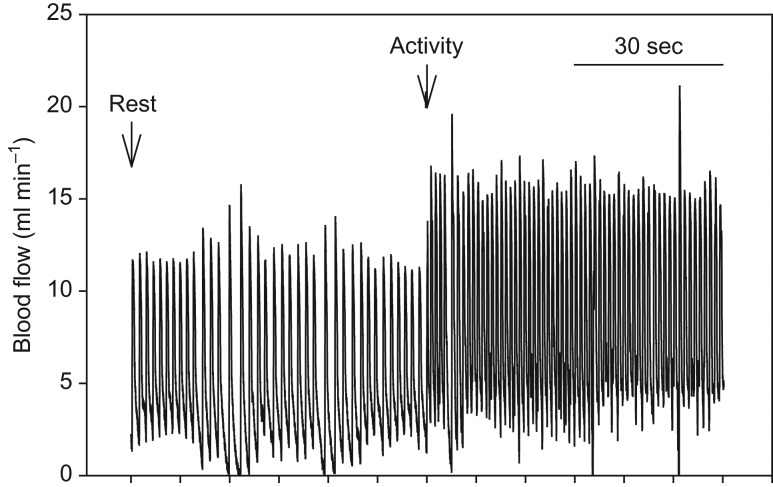
Representative blood flow trace in the ventral aorta during rest and activity for an eel in water at 20°C.

**Figure 4:**
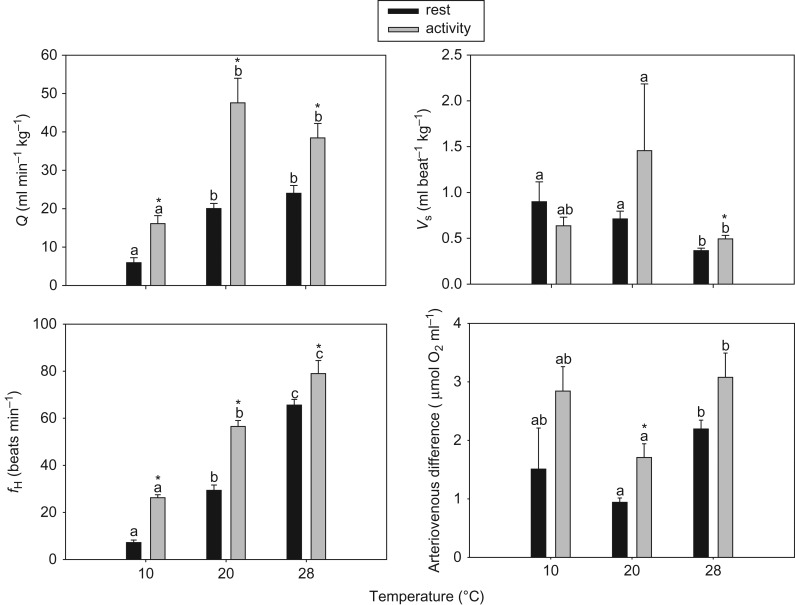
Cardiac output ($\dot{Q}$), heart rate (*f*_H_), stroke volume (*V*_s_) and arteriovenous oxygen concentration difference $\left(C_{{aO}_{2}}-C_{{\bar{v}O}_{2}}\right)$ at different temperatures during rest and activity. *Values are significantly different from each other within temperature groups. Different letters indicate that values for the different temperatures and same treatment (rest or activity) are significantly different from each other. Data are means ± SEM; *n* = 6–8.

As shown in the representative trace for blood flow measured in the ventral aorta at 20°C (Fig. 3), enforced activity caused an immediate and pronounced rise in $\dot{Q}$ and *f*_H_. The $\dot{Q}$ increased significantly in response to activity at all temperatures (*P* < 0.005; one-way repeated-measures ANOVA; Fig. 4) and with temperature (*P* < 0.005 for all significant values; one-way ANOVA; Fig. 4). Heart rate increased significantly from rest to activity at all temperatures (*P* < 0.005; one-way repeated-measures ANOVA; Fig. 4) and increased significantly with temperature during activity and rest (*P* < 0.001; one-way ANOVA; Fig. 4). At 28°C, *V*_s_ varied significantly with treatment (rest and activity). Stroke volume decreased with temperature during rest (*P* = 0.003; one-way ANOVA; Fig. 4).

**Figure 5:**
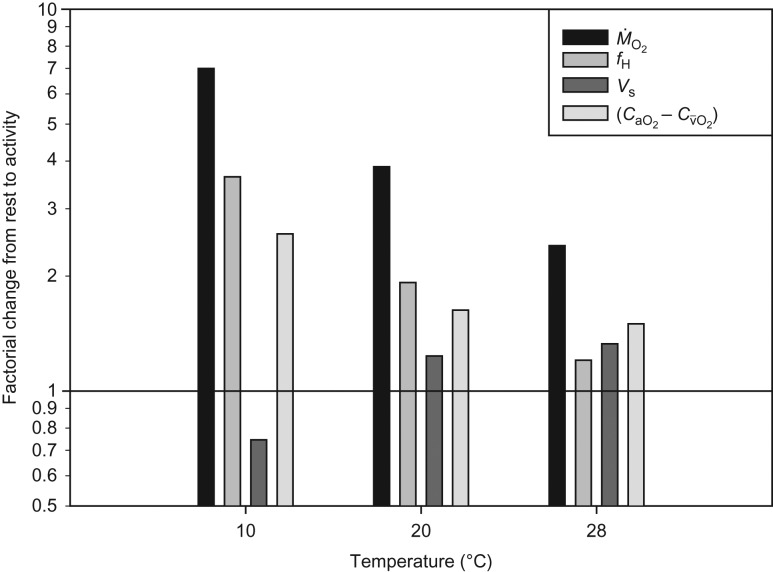
Factorial change in oxygen consumption (${\dot{M}}_{O_{2}}$), heart rate (*f*_H_), stroke volume (*V*_s_) and arteriovenous oxygen concentration difference $\left(C_{{aO}_{2}}-C_{{\bar{v}O}_{2}}\right)$ from rest to activity at different temperatures.

The arteriovenous oxygen difference was calculated from the measurements of $\dot{Q}$ and ${\dot{M}}_{O_{2}}$ performed on two different groups of eels, randomly pooled for statistical purposes. The calculated arteriovenous oxygen difference increased from rest to activity at all temperatures, with the largest extraction occurring at 10°C (Fig. 4). Except at 28°C, *f*_H_ contributed most to the increase in ${\dot{M}}_{O_{2}}$, followed by the arteriovenous difference (Fig. 5).

## Discussion

Our study showed that both SMR and MMR of the European eel increased with acute temperature change, even as temperature approached CT_max_. As a consequence, AS even tended to increase with elevated temperature, and the large AS at temperatures immediately below CT_max_ differs considerably from the ‘bell-shaped’ relationship between AS and temperature proposed in the typical formulation of the OCLTT hypothesis (Pörtner, 2001; Pörtner and Knust, 2007; Pörtner and Farrell, 2008; Farrell, 2009; Nilsson *et al*., 2009; Gardiner *et al*., 2010; Neuheimer *et al*., 2011; Casselman *et al*., 2012). Thus, the persistence of a high AS at temperatures immediately below CT_max_ is not consistent with the OCLTT hypothesis, whereas the smaller increase in *f*_H_ during activity at the high temperatures may be interpreted as an indication of compromised scope for cardiovascular function at high temperatures. In the eels, nevertheless, the low scope for *f*_H_ was compensated by high extraction of oxygen from the arterial blood and high *V*_s_. Thus, even at the high temperatures, the eels are endowed with plenty of scope for oxygen delivery to be allocated for physical activity, digestion, reproduction, etc. The persistence of high AS at temperatures approaching CT_max_ has been reported for a number of fish species (Gollock *et al*., 2006; Mendonça and Gamperl, 2010; Healy and Schulte, 2012; Clark *et al*., 2013; Gräns *et al*., 2014; Norin *et al*., 2014).

The CT_max_ of 31.5°C determined in our experimental protocol closely resembles the lethal temperature of 32°C for eels acclimated to 18°C (Mueller and Nose, 1973), but the eels did not tolerate overnight exposure to 30°C in the respirometers. This probably reflects that the estimated CT_max_ is inversely related to the rate at which temperature is increased during the heating protocol (Pörtner, 2010). Also, the acclimation temperature (the 18°C of the holding tank, in this case) is of paramount importance for the ‘true’ CT_max_. Thus, it is not straightforward to use laboratory findings to predict thermal tolerance in the natural habitat. Nonetheless, it remains clear that the cardiorespiratory systems can provide adequate oxygen transport at CT_max_ in acutely exposed eels. The fact that the eels had plenty of aerobic scope at 28°C, 2°C below the temperature that could not be tolerated overnight, also makes it unlikely that oxygen delivery should form the basis for the tolerance to high temperatures. Consistent with this view, artificial reduction in the haematocrit of sea bass and perch does not appreciably reduce CT_max_ (Wang *et al*., 2014; Brijs *et al*., 2015).

Our intermittent-closed respirometry yielded similar estimates of SMR to those in previous studies on the European eel (McKenzie *et al*., 2000, 2002; Iversen *et al*., 2010; Boldsen *et al*., 2013). We chose chasing to measure ${\dot{M}}_{O_{2}\max}$ because eels normally do not perform prolonged swimming at high speed (Clark *et al*., 2013). The ${\dot{M}}_{O_{2}\max}$ was measured during the immediate period after chasing, as reported in previous studies using this protocol (Norin and Malte, 2011, 2012; Norin *et al*., 2014), although ${\dot{M}}_{O_{2}\max}$ may occur later in other species (Norin and Malte, 2011; Clark *et al*., 2012). On a few occasions, very high ${\dot{M}}_{O_{2}}$ values were recorded during spontaneous activity in the respirometer, indicating that the chasing protocol, like the *U*_crit_ protocol, may not always motivate the fish to reach ${\dot{M}}_{O_{2}\max}$. Nevertheless, our study showed that ${\dot{M}}_{O_{2}\max}$ increased with temperature, and an underestimation of ${\dot{M}}_{O_{2}\max}$ would not alter our conclusions.

Cutaneous gas exchange may contribute up to 35% of SMR in normoxic eels (Berg and Steen, 1965; Kirsch and Nonnotte, 1977). Although primarily devoted to supplying the skin's metabolic rate (Kirsch and Nonnotte, 1977), this uptake will lead to an overestimation of the arteriovenous oxygen concentration difference calculated by the Fick equation (Farrell, 1984). This overestimation is largest at rest because cutaneous oxygen uptake is unlikely to increase during exercise. Therefore, any further exploitation of the venous reserve during exercise is underestimated.

The rise in $\dot{Q}$ with temperature and during activity was primarily mediated through increased *f*_H_ rather than *V*_s_. Numerous other teleosts also elevate blood flow by heart rate responses with small changes in *V*_s_ (e.g. Sandblom and Axelsson, 2007b; Clark *et al*., 2008; Farrell *et al*., 2009). The rise in *f*_H_ with temperature was probably attributable to withdrawal of vagal tone and increased sympathetic tone, which is also likely to have contributed, given the manner of enforcing the activity. During exercise, the reduction in *V*_s_ might be a consequence of shortened cardiac filling time and associated lowering of end-diastolic volume when *f*_H_ increased (e.g. Sandblom and Axelsson, 2007a, b).

### Conclusions and perspectives

The OCLTT model has been presented as a general principle to explain thermal tolerance in aquatic ectotherms, and by linking the cardiorespiratory and mitochondrial functions to thermal tolerance, the OCLTT model provides a mechanistic link to the geographical distribution of animals. Although very appealing, it has proved difficult to establish causality between oxygen limitation and thermal tolerance by experimental manipulation of oxygen delivery and metabolism (Clark *et al*., 2013; Fobian *et al*., 2014; Ern *et al*., 2015; Verberk *et al*., 2016). In this context, the present study also contributes to a steadily increasing number of studies showing that when exposing fish to acute temperature changes, AS does not decrease in a systematic manner as the temperature approaches CT_max_. Thus, in the eels, a number of other teleost fish, as well as other vertebrates and invertebrates, AS continues to rise, or does not decrease, until a few degrees below CT_max_ (Gollock *et al*., 2006; Mendonça and Gamperl, 2010; Healy and Schulte, 2012; Overgaard *et al*., 2012; Clark *et al*., 2013; Ern *et al*., 2014, 2015; Norin *et al*., 2014). Accordingly, there are many examples of animals where compromised oxygen delivery during acute temperature exposure does not link causally to thermal tolerance, and other mechanisms, such as protein denaturation, membrane damage or the uncoupling of biochemical reactions, may be responsible. During temperature acclimation, many changes of importance to the physiology of organisms take place. Whether experimental support for the OCLTT hypothesis becomes more consistent when animals are fully acclimated to the experimental temperatures, however, awaits testing. Thus, at present, we doubt that a unifying theory for upper thermal tolerance can be established, and it is likely that the culprit for upper thermal tolerance differs amongst species.

## Funding

This study was supported by the The Danish Council for Independent Research | Natural Sciences.
